# Latent classes of adolescent health behaviour, social covariates and mental wellbeing: a longitudinal birth cohort study

**DOI:** 10.1186/s12889-024-20004-y

**Published:** 2024-09-18

**Authors:** Christopher Knowles, Emma Thornton, Kimberly Petersen, Suzet Tanya Lereya, Neil Humphrey

**Affiliations:** 1 Manchester Institute of Education, University of Manchester, Manchester, M15 6JA UK; 2 School of Education, University of Leeds, Leeds, M15 6JA UK; 3 Evidence Based Practice Unit (EBPU), University College London and Anna Freud, London, N1 9JH UK; 4 Ellen Wilkinson Building, University of Manchester, Oxford Road, Manchester, M15 6JA UK

**Keywords:** Adolescents, Diet, Health behaviour, Physical activity, Sleep, Mental Wellbeing

## Abstract

**Background:**

Adolescent mental wellbeing has been declining in the United Kingdom for over a decade. Expansion of services to support the mental wellbeing of young people is a public health priority and a core component of the National Health Service’s Long-Term Plan. In this paper, we leverage secondary analysis of a very large longitudinal dataset (#BeeWell) to generate insights regarding different patterns of health behaviour, their covariates, and consequences for mental wellbeing one year later.

**Methods:**

A Latent Class Analysis was conducted using data on physical activity, sleep, and eating habits collected in 2021 from 18,478 Year 8 pupils from Greater Manchester (United Kingdom) to (1) identify distinct latent classes of adolescent health behaviour; (2) establish factors likely to be associated with latent class membership; and (3) determine whether latent class membership contributes to variance in self-reported mental wellbeing one year later.

**Results:**

A three-class solution was identified as an excellent fit to the data, discriminating between: the *Wellness Weary* (*n* = 2,717; 15%); the *Balanced Bunch* (*n* = 7,377; 40%); and the *Green and Dream Team* (*n* = 8,384; 45%). Several factors significantly influenced class membership. Most notably, socio-economic disadvantage and social media use were linked with less favourable health behaviour patterns, whilst cisgender heterosexual girls were likely to endorse healthier patterns. After adjusting for covariates, the *Green and Dream Team* reported significantly greater mental wellbeing than the *Balanced Bunch* one year later. However, there was no difference between the *Balanced Bunch* and the *Wellness Weary*, signalling that health behaviours may support mental wellbeing, but only among the healthiest young people.

**Conclusions:**

Beyond advancements in fundamental understanding, our findings yield significant translation opportunities through their use and application in health, education, and allied professional settings designed to support young people.

**Supplementary Information:**

The online version contains supplementary material available at 10.1186/s12889-024-20004-y.

## Background

Health behaviours are a set of practices that promote or impair the health of an individual [1]. Adolescence is a transformative phase of life, and a critical developmental period characterised by rapid neurological, psychosocial, and emotional development [2]. Many young people start to develop their own ideas and opinions of different health behaviours during this time. Although parents and other role models may still influence the decision to engage in certain behaviours, those endorsed during this key life stage can become habitual and persist into early adulthood [3]. *Physical activity* [4], *sleep* [5], and *eating habits* [6], previously coined *The Big Three* modifiable health behaviours, are proposed to have both independent and synergistic associations with adolescent mental health [7, 8]. Theoretical models of these associations span neurobiological, psychosocial, and behavioural processes [9]. For example, physical activity impacts on the functioning of the hypothalamus-pituitary-adrenal axis, which in turn reduces cortisol levels, thereby supporting mental wellbeing [10]. Inadequate sleep can prompt more frequent use of maladaptive emotion regulation strategies, which in turn negatively impacts mental health [11].

A great number of factors may influence the likelihood an individual endorses different health behaviours in adolescence [12] including ethnicity [13–15]; socio-economic disadvantage [16–18]; social media use [19, 20]; physical health [13]; and prior levels of mental wellbeing (for which the relationship is likely reciprocal) [21–23]. Some less well understood covariates of health behaviour endorsement may also hold particular relevance for the adolescent population including gender identity and sexual orientation [24, 25], and bullying victimisation.

There is evidence that gender and sexual minority groups are more likely to be physically inactive, possibly due to reluctance to use communal changing facilities, or the stigma that exists around sexuality in sport. LGBTQ+ young people have also been observed as more likely to engage in maladaptive eating habits [24, 25] which can emerge as coping mechanisms in response to the heightened stress these young people face. Evidence for the sleep health of LGBTQ+ young people is inconsistent but potentially points to an unmet need for support hence, merits further research [26].

Although the direction of causality is unclear (and indeed, the relationship might be reciprocal), studies have observed a negative correlation between eating habits and bullying [27]. Similarly, there is some evidence for a reciprocal relationship between bullying and physical activity in adolescence [28]. Failure to meet physical activity guidelines can lead to bullying due to poor motor skill development, low physical fitness and low self-confidence to engage in certain activities [29]. Bullying may also lead to inactivity as a method of self-preservation by avoiding potentially embarrassing situations if a young person perceives themselves to have low level competency in the activity being performed. Better understanding of these factors can help identify young people at-risk and contribute to effective, targeted behaviour change interventions to support their mental wellbeing.

There is now widespread evidence that being sufficiently active, getting enough sleep, and following a healthy diet can support mental health in adolescence [7]. From a population health perspective, a focus on mental wellbeing arguably has greater utility than a focus on mental illness [30]. Most young people do not meet diagnostic criteria for a mental health disorder (leading to floor effects), but there is substantial variability in mental wellbeing [31], which has been demonstrated to predict a range of salient outcomes later in life including but not limited to: adult mental and physical health; health behaviours; relationships; and labour market outcomes [32].

### A person-centred approach

Previous studies in this area have tended to adopt variable-centred approaches to analysis (e.g., regression) which assume a homogeneous population differing only in the extent to which it engages in health behaviours. In comparison, relatively few studies adopt person-centred perspectives (e.g., cluster or latent class/profile analysis) which aim to capture the heterogeneity that exists within populations in terms of the extent *and* pattern of health behaviours they exhibit [33]. Where variable-centred approaches examine associations between variables, person-centred approaches examine relationships between people, offering evidence of how certain health behaviours might cluster together in distinct patterns that characterise unobserved subgroups (i.e., latent classes) of the population [33, 34]. Each latent class comprises individuals who elicit similarities on specific indicators, but who are quantitatively and qualitatively distinct from those in alternative classes, thus capturing homogeneity within-, and heterogeneity between-groups [34]. Given that adolescence can be a particularly transformative phase of life: the extent to which young people endorse different health behaviours; the effect of social and demographic antecedents of health behaviour; and the collective impact health behaviours may have on mental wellbeing is likely to differ significantly from one individual to the next [2]. Person-centred research is therefore, both warranted and necessary in this age-group.

A useful illustrative example of the utility of person-centred approaches is seen in a cross-sectional study [35], which used cluster analysis to identify three distinct patterns of health behaviour (utilising data on sleep, alcohol use, cannabis use, social media use, and sport and hobby participation) among Irish adolescents, denoted as low, moderate and high health-promoting, respectively. The authors found that membership of these clusters was predicted by socio-demographic characteristics (e.g., high health-promoting adolescents were likely to be younger and female); they also reported that cluster membership was associated with outcomes pertaining to mental wellbeing (e.g., low health-promoting adolescents reported the highest levels of anxiety and depression, and lowest levels of life satisfaction). Whilst extremely illuminative, existing person-centred studies of adolescent health behaviour have predominantly been cross-sectional [35–38], focused on a single health behaviour rather than clusters of behaviours, [37], have elected to focus on mental illness as opposed to wellbeing or outcomes beyond mental health altogether [36–39]. There is an ongoing need to establish temporal precedence between *collective patterns of health behaviour* and adolescent mental wellbeing both before and after adjusting for a wide range of social and demographic covariates.

### Aims and hypotheses

Using data from the first two annual waves (T1, T2) of the #BeeWell study in Greater Manchester, United Kingdom, the aims of this study were to establish: (1) latent classes of adolescent health behaviour at T1 when participants were aged 12-13; (2) whether bullying victimisation, social media use, gender identity and sexual orientation, ethnicity, socio-economic disadvantage, self-reported physical health, and/or mental wellbeing at T1 were associated with latent class membership; and, (3) whether latent class membership at T1 contributed to variance in mental wellbeing at T2, when participants were aged 13-14 years.

The class identification phase of analysis was largely exploratory in nature. We hypothesised there may be a predominantly healthy class (e.g., physically active, sufficient sleep, regular fruit and vegetables, irregular confectionary) and a predominantly unhealthy class (e.g., physically inactive, insufficient sleep, irregular fruit and vegetables, regular confectionary), among others (H1) [37]. We further hypothesised, based on existing evidence noted above, that bullying victimisation, LGBTQ+ adolescents, minority ethnicity, socio-economic disadvantage, poor physical health, and poor T1 mental wellbeing would be significant risk factors for membership of less healthy classes (H2). Finally, we hypothesised that members of more healthy classes would report better mental wellbeing at T2 after controlling for covariates (H3). An analysis plan detailing our hypotheses and analytical methods was pre-registered on the Open Science Framework [40].

## Methods

### Participants

#BeeWell is a hybrid population cohort study comprising: (i) a truncated longitudinal study in which participants are tracked with annual data points from age 12-15 (i.e., from Year 8 to Year 9 to Year 10 of secondary school; Sample 1); and, (ii) a cross-sectional study comprising annual data points for participants aged 14-15 (i.e., those in Year 10 of secondary school at a given data point; Sample 2) [41]. Our secondary analysis drew on the first and second annual data points for Sample 1 in Greater Manchester conducted in 2021 (T1) and 2022 (T2), respectively (overall *N* = 20,241).

All young people from Sample 1 who responded to at least one of the health behaviour items at T1 were eligible for inclusion. Data were clustered by school, so consistent with guidance for working with multilevel data, only cases were there were ≥ 5 pupils per school were retained [42]. This resulted in a final analytical sample of *n* = 18,478 pupils from 138 schools. Supplementary Material has been provided to facilitate comparison of the demographic characteristics of the analytical sample against those for Greater Manchester and England. In brief, the composition of the analytical sample largely mirrors the Greater Manchester population from which it is drawn in terms of sex, ethnicity, having English as an additional language, and special educational need however, contains fewer young people eligible for free school meals. Similarly, the analytical sample largely mirrors the national population of England in terms of sex, free school meal eligibility, having English as an additional language and special educational need, but contains a somewhat lower proportion of White British young people than seen nationally.

### Measures

#BeeWell is a rich source of individual-level data on adolescent mental wellbeing (e.g., life satisfaction, self-esteem, negative affect), covariates of positive mental wellbeing (e.g., social media use, bullying, sleep), and sociodemographic characteristics (e.g., age, ethnicity, sexual orientation). Data pertaining to latent class indicators (physical activity, sleep, fruit and vegetable consumption, confectionary consumption) and covariates (ethnicity, socio-economic disadvantage, gender identity, sexual orientation, self-reported physical health, social media use, bullying victimisation and mental wellbeing) were drawn from the T1 survey and linked administrative data provided by Greater Manchester Local Authorities. Outcome data on mental wellbeing were drawn from the T2 survey. A detailed explanation of the measures used and how scores were interpreted is provided in Table 1. The #BeeWell survey can also be accessed online [43].

**Table 1:** Sample characteristics and measures used to quantify adolescent health behaviour, covariates and mental wellbeing

Variable	Measure	Description		Score
Latent Class Indicators				
Fruit and Vegetable Consumption *(missing = 0.49%)*	Single item adapted from the Health Behaviours in Schools Checklist [44] and the Millennium Cohort Study [45] targeting weekly consumption of fruit and vegetables.	Responses treated as quasi-continuous, rated on a seven-point scale ranging from 0 (*Never*) to 6 (*Everyday more than once*).	Mean (S.D.)	5.06 (1.60)
Confectionary Consumption *(missing = 0.71%)*	Single item adapted from the Health Behaviours in Schools Checklist [44] and the Millennium Cohort Study [45] weekly consumption of sweets, chocolate, crisps, and fizzy drinks.	Responses treated as quasi-continuous, rated on a seven-point scale ranging from 0 (*Never*) to 6 (*Everyday more than once*). Reverse coded such that higher scores represented healthier behaviour.	Mean (S.D.)	3.24 (1.44)
Physical Activity *(missing = 3.44%)*	Two items adapted from the Health Behaviours in Schools Checklist [44] measuring weekly minutes of Moderate-to-Vigorous Physical Activity (MVPA).	A binary variable was derived discriminating between participants adhering/not adhering to current CMO MVPA guidelines (≥420-minutes per week). Responses coded: *No* = 0; *Yes* = 1.	*Yes* *No*	n (%) 6,590 (35.66%) 11,253 (60.90%)
Sleep *(missing = 0.87%)*	A single item from the Health Behaviours in Schools Checklist asking whether the amount of sleep they normally get is sufficient to feel awake and concentrate on schoolwork during the day [44].	Responses coded: *No* = 0; *Yes* = 1.	*Yes* *No*	n (%) 11,732 (63.49%) 6,586 (35.64%)
Covariates of Latent Class Membership				
Mental Wellbeing *(missing = 11.69%)*	The (seven-item) Short Warwick-Edinburgh Mental Wellbeing scale (SWEMWBS) [46].	A five-point Likert-type scale. Total scores range from 7 to 35 with higher scores indicating greater mental wellbeing. Consistent with recommendations from the scale developer, *transformed* SWEMWBS scores were used [47].	Mean (S.D.)	21.75 (4.95)
Bullying Victimisation *(missing = 5.31%)*	Three items adapted from the Understanding Society Youth Questionnaire [47] and the Health Behaviours in Schools Checklist [44].	A binary measure of bullying was derived. Participants who responded *quite a lot* or *a lot* to at least one item were classed as bullied. Coded as: *Not Bullied* = 0; *Bullied* = 1.	*Bullied* *Not bullied*	n (%) 3,092 (16.73%) 14,405 (77.96%)
Ethnicity *(missing = 3.88%)*	Classed as Asian, Black, Mixed, White, or Any Other Ethnic Group (including Chinese) using linked administrative data provided by Greater Manchester Local Authorities.	Dummy variables derived for each ethnic group. Coded as: *No* = 0; *Yes* = 1.	*Asian* *Black* *Mixed* *White* *AOEG*	n (%) 3,190 (17.26%) 903 (4.89%) 1,077 (5.83%) 11,998 (64.93%) 593 (3.21%)
Self-Reported Physical Health *(missing = 0.23%)*	A single item adapted from Understanding Society [48].	A five-point Likert-type scale ranging from 1 (*Poor*) to 5 (*Excellent*).	*Mean (S.D.)*	2.42 (1.022)
Gender Identity and Sexual Orientation *(missing = 7.33%)*	A three-category variable was derived using sex assigned at birth (linked administrative data), gender identity and sexual orientation (gathered through T1 surveys) [49].	Cisgender Heterosexual Boys (reference group); Cisgender Heterosexual Girls; and LGBTQ+. Dummy variables derived for each category. Coded as: *No* = 0; *Yes* = 1.	*Cishet Boy* *Cishet Girl* *LGBTQ+*	n (%) 6,215 (33.63%) 5,366 (29.04%) 5,542 (29.99%)
Social Media Use *(missing = 5.84%)*	A single item adapted from the Millennium Cohort Study assessing daily hours spent on social media [45].	A continuous variable whereby higher scores represent more frequent daily use (hours).	Mean (S.D.)	4.29 (2.51)
Socio-Economic Disadvantage *(missing = 3.36%)*	Index of Multiple Deprivation (IMD) rank based on the Lower Layer Super Output Area (LSOA) for the young person’s home postcode (provided by Greater Manchester Local Authorities) ranging from 1 (*Most Deprived*) to 32,844 (*Least Deprived*).	The reciprocal of IMD rank expressed as a percentage was used such that scores ranged from 0 to 1 and higher scores indicated greater disadvantage.	Mean (S.D.)	.65 (.30)
Outcomes of Latent Class Membership				
Mental Wellbeing *(missing = 46.23%)*	The (seven-item) Short Warwick-Edinburgh Mental Wellbeing scale (SWEMWBS) [46].	A five-point Likert-type scale. Total scores range from 7 to 35 with higher scores indicating greater mental wellbeing. Consistent with recommendations from the scale developer, *transformed* SWEMWBS scores were used [47].	Mean (S.D.)	21.78 (5.04)

### Statistical methods

**Fig. 1:**
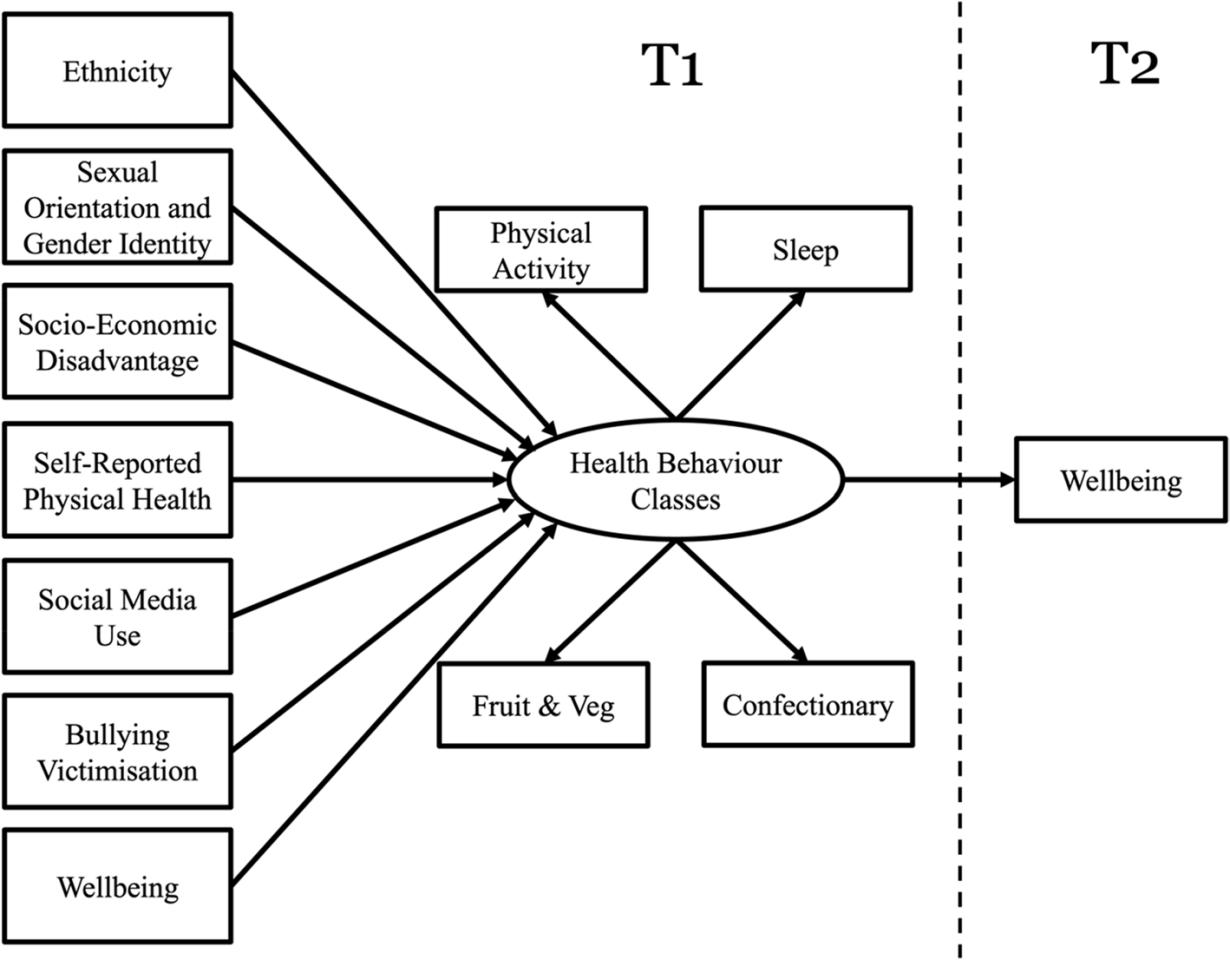
Conceptual framework outlining the study design and timepoint at which each variable was measured

All analyses were conducted using Mplus version 8.9 [50]. A Maximum Likelihood Three-Step Approach was used [51]. This method adopts a stepwise approach wherein the optimal latent class model is identified without the inclusion of covariates, using a range of model fit statistics (step one). Participants are then assigned to their most likely latent class, while accounting for classification error using average posterior probabilities (step two). Finally, covariates and distal outcomes of latent class membership are included in the final latent class regression model as auxiliary variables (step three). A conceptual framework for the current study is illustrated in Fig. 1. Full Information Maximum Likelihood (FIML) was used to handle missing data [52], details of which can be found in column one of Table 1. To investigate whether missing data posed a risk of bias, a complete-case sensitivity analysis was conducted whereby missing data were removed listwise. Results of the sensitivity analysis are provided as Supplementary Material.

### Latent class enumeration

Latent class analysis was conducted using four health behaviours as class indicators (physical activity; sleep sufficiency; fruit and vegetable consumption; confectionary consumption). As data were clustered by school, a sandwich estimator was used (in Mplus ‘type=complex’). To identify the most optimal number of classes, starting with a one-class solution, models with a consecutive number of latent classes were run until convergence problems were encountered. The following model fit statistics were consulted to determine which solution offered the best fit to the data: *Akaike Information Criteria* (AIC); *Bayesian Information Criteria* (BIC); and *Sample Size Adjusted Bayesian Information Criteria* (ssaBIC), for which lower values indicate better model fit [53]. *Lo-Mendell-Rubin Adjusted Likelihood Ratio Tests* (LMRa) significant at *p* < .05 suggested a model was a significantly better fit to the data than that containing one less latent class (i.e., *k* vs. *k* –1) [54]. Classification entropy is not a fit index thus, was not used in the class enumeration process [55]. Nevertheless, entropy values are reported with values ≥ .80 considered to represent acceptable levels of classification accuracy [56].

To avoid overfitting the model to the data and diluting generalisability of findings, quantitative fit statistics were considered alongside substantive criteria such as the interpretability of the classes, model parsimony (with the simplest solution preferred), and the proportional distribution of the sample (i.e., models with very small classes were considered unstable) [57]. Lastly, to strengthen reliability of the class enumeration process, a split halves analysis was conducted whereby the analytical sample was randomly split in half and the class enumeration process repeated in each to determine whether the best fitting model was consistent throughout [58].

### Latent class regression analysis and mean difference tests

Once the most optimal latent class model was identified (H1) the maximum likelihood three-step method [51] was used to simultaneously include covariates and distal outcomes in the model. To determine whether covariates were predictive of class membership (H2), a single multinomial logistic regression was conducted whereby class membership was regressed on all covariates. Wald tests of mean difference tests were conducted to establish statistically significant differences in mental wellbeing at T2 (H3). This was first assessed using an unadjusted model, followed by a partially adjusted model controlling for baseline mental wellbeing, and a third fully adjusted model which controlled for all covariates (Fig. 1). Multiple reference groups were used to facilitate comparisons in wellbeing between all latent classes.

## Results

### Class enumeration

**Fig. 2:**
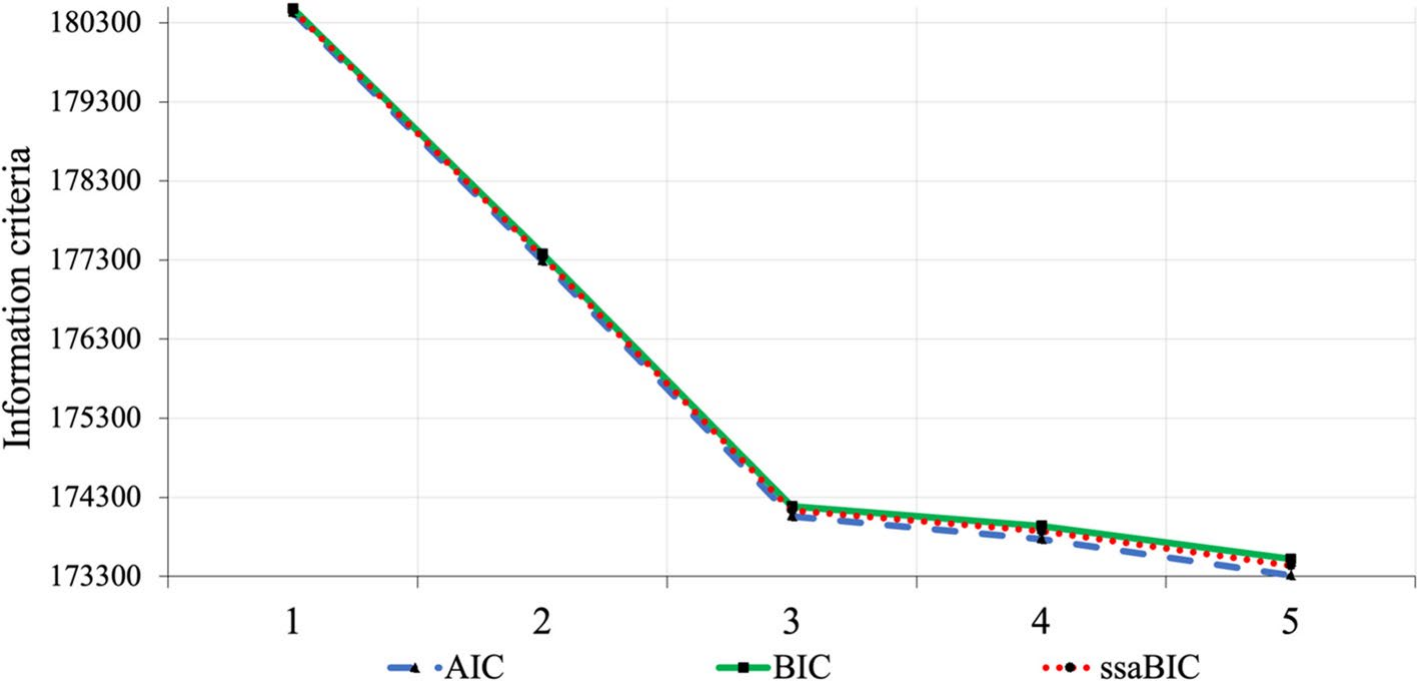
Elbow plot illustrating latent class solutions for patterns of adolescent health behaviour

Convergence issues arose from the six-class solution onward. Accordingly, fit statistics for the one- to five-class solutions are presented in Table 2. Information criteria-based fit statistics are also illustrated as an elbow plot to aid interpretation (Fig. 2). A clear elbow is visible at the three-class solution indicating that beyond this point, increasing model complexity yielded diminishing returns in model fit. LMRa results inferred the *k*-solution was a better fit in every instance however, the four- and five-class solutions contained very small latent classes (6% and 3% of the sample, respectively). Conversely, the smallest class for the three-class solution contained 15% of the sample; well above the guideline threshold of 10% recommended to avoid over-extraction [57]. Split halves analysis reinforced findings of the main class enumeration; identifying a three-class model as the best fit to the data (see Supplementary Material). In summation, the three-class solution was considered the most quantitatively and qualitatively parsimonious model and was advanced for further analysis. Entropy for the three-class solution was .916, indicating excellent classification accuracy.

**Table 2:** Model fit statistics for latent classes of adolescent health behaviours

Classes	LL	AIC	BIC	ssaBIC	LMRa	Entropy	Model Estimated Class Proportions
1	-90210.016	180432.031	180478.977	180459.910	-	-	1
2	-88634.063	177290.125	177376.193	177341.236	.000	.729	.58, .42
3	-87014.105	174060.209	174185.399	174134.552	.000	.916	.45, .40, .15
4	-86865.065	173772.131	173936.442	173869.705	.000	.895	.45, .40, .08, .06
5	-86631.204	173314.408	173517.841	173435.214	.000	.833	.45, .27, .16, .07, .03

*AIC* Akaike Information Criteria, *BIC* Bayesian Information Criteria, *LL* Loglikelihood, *LMRa* Lo-Mendell-Rubin Adjusted Likelihood Ratio Test, *ssaBIC* Sample Size Adjusted Bayesian Information Criteria

### Class structure

**Fig. 3:**
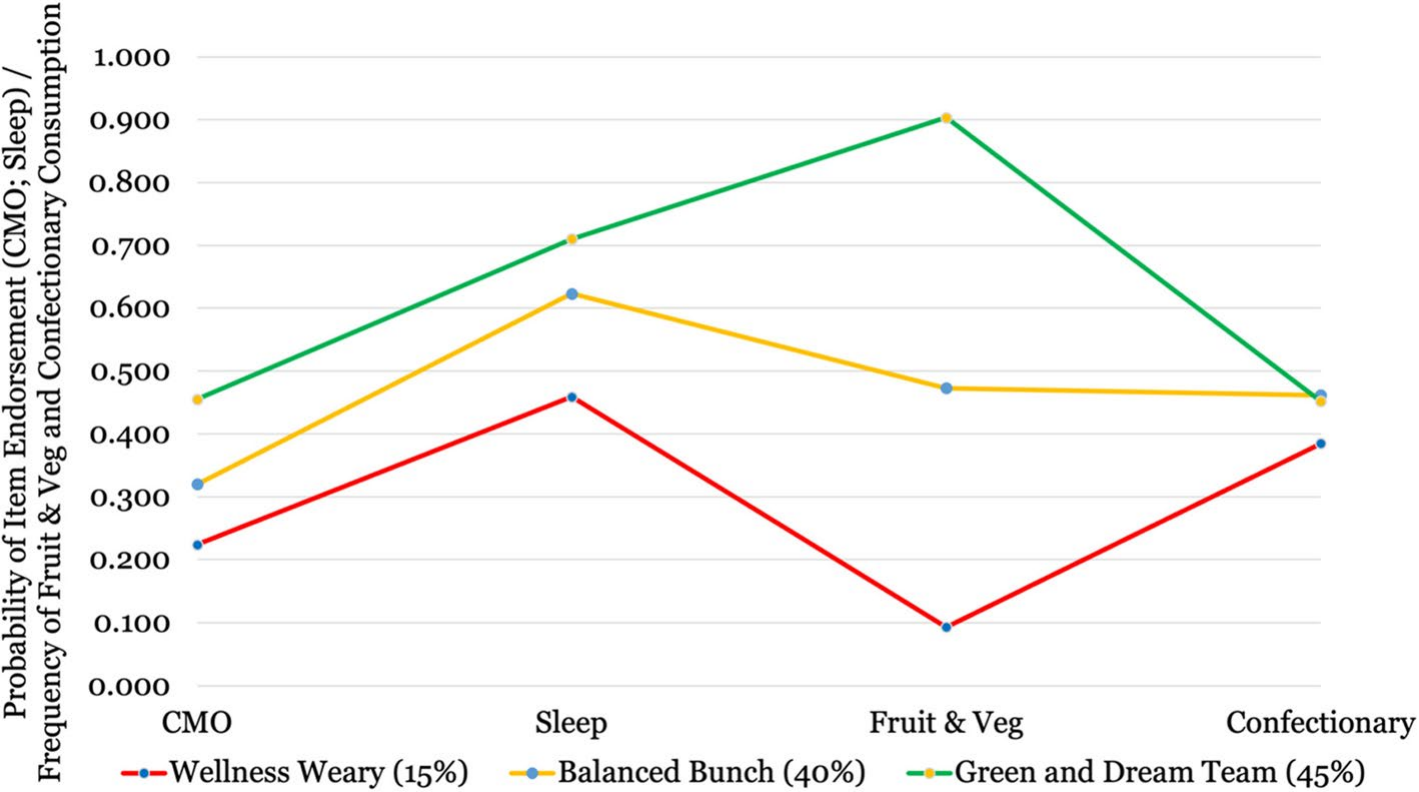
Probability plot illustrating the three latent classes of adolescent health behaviour. Y-axis reflects endorsement of CMO guidelines (probability), getting sufficient sleep (probability), frequency of fruit and vegetable (percentage) and confectionary consumption (percentage) Note: When using differently scaled indicators, some data manipulation is necessary to generate an easily interpretable figure with all latent class indicators presented in one plot. Class-specific mean scores for fruit/veg and confectionary consumption were converted to a percentage (with confectionary reverse coded so higher scores represent healthier outcomes in all instances) meaning indicators are all scored on an equivalent scale of 0-1 (e.g., response categories ranged from 0 to 6, so a score of 3 = .50). Original item-responses were maintained for use in statistical analysis. See Table 1 for more details

The structure of the three latent classes is illustrated in Fig. 3. Descriptive information for the proportional distribution of the sample and class-specific mean values for each of the latent class indicators and covariates are provided in Table 3. The red class, henceforth referred to as the *Wellness Weary* (*n* = 2,717, 14.7%)*,* were least likely to be active or get sufficient sleep and ate fruit and vegetables relatively infrequently. Compared to the rest of the sample, the amber class, the *Balanced Bunch* (*n* = 7,377, 39.9%)*,* exhibited a moderate likelihood of being active, getting sufficient sleep and eating fruit and vegetables. The green class, the *Green and Dream Team* (*n* = 8,384, 45.4%) were most likely to be active, get sufficient sleep, and ate substantially more fruit and vegetables than the other classes. Confectionary consumption was largely homogeneous across all latent classes, although the *Wellness Weary* did consume sweets, chocolate, crisps and fizzy drinks slightly more often than the other two classes.

**Table 3:** Proportional distribution of the sample and observed mean values for participants who provided data on each variable

		Wellness Weary (*n* = 2,717)			Balanced Bunch (*n* = 7,377)			Green and Dream Team (*n* = 8,384)		
Variable	Metric	Valid *n*	Missing *n* (%)	Mean/Proportion	Valid *n*	Missing *n* (%)	Mean/Proportion	Valid *n*	Missing *n* (%)	Mean/Proportion
Meet Physical Activity Guidelines	n (%)	2,562	155 (5.7)	597 (22.0)	7,147	230 (3.1)	2,306 (31.3)	8,134	250 (3.0)	3,687 (44.0)
Get Sufficient Sleep	n (%)	2,687	30 (1.1)	1,287 (47.4)	7,319	58 (0.8)	4,542 (61.6)	8,134	72 (0.9)	5,903 (70.4)
Fruit and Vegetable Consumption	Mean (S.D.)	2,717	0 (0.0)	1.49 (.500)	7,355	22 (0.3)	3.405 (.491)	8,316	68 (0.8)	5.537 (.499)
Confectionary Consumption	Mean (S.D.)	2,691	26 (1.0)	2.976 (1.625)	7,330	47 (0.6)	3.30 (1.317)	8,325	59 (0.7)	3.255 (1.424)
Bullied	n (%)	2,540	177 (6.5)	591 (21.8)	6,961	416 (5.6)	1,207 (16.4)	7,996	388 (4.6)	1,294 (15.4)
Asian Ethnicity	n (%)	2,622	95 (3.5)	522 (19.2)	7,056	321 (4.4)	1,290 (17.5)	7,908	476 (5.7)	1,378 (16.4)
Black Ethnicity	n (%)	2,622	95 (3.5)	208 (7.7)	7,056	321 (4.4)	367 (5.0)	7,908	476 (5.7)	328 (3.9)
Mixed Ethnicity	n (%)	2,622	95 (3.5)	132 (4.9)	7,056	321 (4.4)	462 (6.3)	7,908	476 (5.7)	483 (5.8)
AOEG	n (%)	2,622	95 (3.5)	51 (1.9)	7,056	321 (4.4)	137 (1.9)	7,908	476 (5.7)	230 (2.7)
Cisgender Heterosexual Girl	n (%)	2,500	217 (8.0)	686 (25.2)	6,827	550 (7.5)	2,151 (29.2)	7,796	588 (7.0)	2,529 (30.2)
LGBTQ+	n (%)	2,500	217 (8.0)	1,038 (38.2)	6,827	550 (7.5)	2,151 (29.2)	7,796	588 (7.0)	2,353 (28.1)
Physical Health	Mean (S.D.)	2,711	6 (0.2)	2.849 (1.126)	7,368	9 (0.1)	2.512 (.981)	8,356	28 (0.3)	2.198 (.964)
Index of Multiple Deprivation	Mean (S.D.)	2,651	66 (2.4)	.724 (.262)	7,153	224 (3.0)	.668 (.287)	8,053	331 (3.9)	.600 (.309)
Social Media Use	Mean (S.D.)	2,549	170 (6.3)	5.111 (2.650)	6,893	484 (6.6)	4.531 (2.447)	7,958	426 (5.1)	3.821 (2.412)
Baseline Mental Wellbeing	Mean (S.D.)	2,335	382 (14.1)	19.964 (4.808)	6,458	919 (12.5)	21.467 (4.650)	7,525	859 (10.2)	22.551 (5.063)

Statistics represent probable distribution of latent classes and are not to be treated as absolute values. Values presented above are based on participants with valid data on each item. Full information maximum likelihood estimation and posterior probabilities were used to handle missing data and account for classification error, respectively

*AOEG* Any Other Ethnic Group, *LGBTQ*+ Lesbian Gay Bi-Sexual Transgender Queer

*Metric* The metric used to quantify class-specific scores (mean) and sample distribution (proportion) for each variable

### Covariates of health behaviour class membership

All covariates were measured at T1 (see Table 1 for more details). For all class comparisons, *Wellness Weary* were used as a reference hence, covariates with Odds Ratios > 1.00 should be considered factors that increased the likelihood adolescents endorsed healthier patterns of behaviours. Results are presented in Table 4. In brief, members of healthier classes were significantly more likely to be cisgender heterosexual girls (*Balanced Bunch* [OR=1.191]; *Green and Dream Team* [OR=1.466]), have better baseline mental wellbeing (*Balanced Bunch* [OR=1.041]; *Green and Dream Team* [OR=1.063]), and more favourable self-perceived physical health (*Balanced Bunch* [OR=1.225]; *Green and Dream Team* [OR=1.587]). Healthier classes were also significantly less likely to be socioeconomically disadvantaged (*Balanced Bunch* [OR=.553]; *Green and Dream Team* [OR=.276]), of Asian (*Balanced Bunch* [OR=.812]; *Green and Dream Team* [OR=.756]) or Black (*Balanced Bunch* [OR=.586]; *Green and Dream Team* [OR=.487]) ethnicity, and likely spent less time using social media (*Balanced Bunch* [OR=.929]; *Green and Dream Team* [OR=.841]). The *Balanced Bunch* were also significantly more likely to be of Mixed ethnicity (OR=1.277) and less likely to identify as LGBTQ+ (OR=.814).

**Table 4:** Results of multinomial logistic regression analysis conducted to establish covariates of health behaviour class membership

			95% CI					95% CI	
Variable	Class	OR (S.E.)	Lower	Upper	Variable	Class	OR (S.E.)	Lower	Upper
Physical Health	WW	1	1	1	Mixed Ethnicity	WW	1	1	1
	BB	1.225 (.037)^c^	1.155	1.300		BB	1.277 (.138)^a^	1.033	1.580
	GDT	1.587 (.051)^c^	1.491	1.690		GDT	1.226 (.132)	.993	1.513
Index of Multiple Deprivation	WW	1	1	1	AOEG	WW	1	1	1
	BB	.553 (.061)^c^	.445	.686		BB	.971 (.195)	.655	1.440
	GDT	.276 (.038)^c^	.210	.361		GDT	1.529 (.359)	.965	2.422
Social Media	WW	1	1	1	Cisgender Heterosexual Girl	WW	1	1	1
	BB	.929 (.011)^c^	.908	.950		BB	1.191 (.088)^a^	1.030	1.377
	GDT	.841 (.010)^c^	.821	.861		GDT	1.466 (.118)^c^	1.252	1.716
Bullied	WW	1	1	1	LGBTQ+	WW	1	1	1
	BB	.912 (.061)	.800	1.040		BB	.814 (.057)^b^	.710	.934
	GDT	1.055 (.072)	.923	1.206		GDT	.954 (.070)	.826	1.101
Asian Ethnicity	WW	1	1	1	Baseline Mental Wellbeing	WW	1	1	1
	BB	.812 (.058)^b^	.705	.934		BB	1.041 (.007)^c^	1.028	1.054
	GDT	.756 (.061)^b^	.645	.886		GDT	1.063 (.007)^c^	1.050	1.076
Black Ethnicity	WW	1	1	1					
	BB	.586 (.081)^c^	.446	.769					
	GDT	.487 (.064)^c^	.376	.630					

White ethnicity and cisgender heterosexual boys were reference categories hence, were not analysed to avoid multicollinearity

*AOEG* Any Other Ethnic Group, *BB* Balanced Bunch, *GDT* Green and Dream Team,  *LGBTQ+* Lesbian Gay Bi-Sexual Transgender Queer, *WW* Wellness Weary

^a^Significant at the .05 level

^b^Significant at the .01 level

^c^Significant at the .001 level

### Later mental wellbeing as an outcome of health behaviour class membership

Results are presented in Table 5. All between-class differences in mental wellbeing at T2 were tested within each model. Analysis adopted a hierarchical structure composed of an unadjusted, partially adjusted and fully adjusted model. Before adjusting for covariates, mental wellbeing scores one year later differed significantly across all latent classes, increasing from the *Wellness Weary* to the *Balanced Bunch* (Mean Difference = .813(.196), *p* < .001, *d* = .162), and the *Balanced Bunch* to the *Green and Dream Team* (Mean Difference = .900(.117), *p* < .001, *d* = .180). After adjusting for baseline mental wellbeing, the difference between *Wellness Weary* and the *Balanced Bunch* became non-significant (Mean Difference = .074(.179), *p =* .682, *d* = .016) but differences between the *Wellness Weary* and the *Green and Dream Team* (Mean Difference = .426(.190), *p* = .025, *d* = .095) and the *Balanced Bunch* and the *Green and Dream Team* (Mean Difference = .352(.103), *p* = .001, *d* = .079) remained. In the fully adjusted model, an effect remained whereby the *Green and Dream Team* reported significantly greater T2 mental wellbeing than the *Balanced Bunch* (Mean Difference = .219(.098), *p* = .026, *d* = .050). No other significant differences were observed. In the complete-case sensitivity analysis, this difference became non-significant however, we attribute this inconsistency to the reduction in sample size (*n* = 18,478 to *n* = 13,683) and the full-case models’ diminished power to detect significant effects (see Supplementary Material).

**Table 5:** Results of wald mean difference tests comparing mental wellbeing at T2 between all identified classes

	Unadjusted Model			Partially Adjusted Model^a^			Fully Adjusted Model^b^		
Comparison	Mental Wellbeing	MD (S.E.)	*d*	Mental Wellbeing	MD (S.E.)	*d*	Mental Wellbeing	MD (S.E.)	*d*
WW	20.637 (.176)	ref	-	21.478 (.171)	ref	-	21.642 (.168)	ref	-
BB	21.450 (.113)	.813 (.196)^***^	.162	21.551 (.105)	.074 (.179)	.016	21.560 (.101)	-.082 (.178)	-.019
GDT	22.350 (.125)	1.713 (.212)^***^	.342	21.904 (.118)	.426 (.190)^*^	.095	21.779 (.117)	.138 (.190)	.032
BB	21.450 (.113)	ref	-	21.551 (.105)	ref	-	21.560 (.101)	ref	-
GDT	22.350 (.125)	.900 (.117)^***^	.180	21.904 (.118)	.352 (.103)^**^	.079	21.779 (.117)	.219 (.098)^*^	.050

*BB* Balanced Bunch*,* *GDT* Green and Dream Team*,* *MD* Mean Difference, *WW* Wellness Weary

^*^Significant at the .05 level

^**^Significant at the .01 level

^***^Significant at the .001 level

^a^Adjusted for baseline mental wellbeing

^b^Adjusted for baseline mental wellbeing, physical health, IMD, social media use, bullying victimisation, ethnicity, gender identity and sexual orientation

## Discussion

The purpose of this study was to elucidate patterns of adolescent health behaviour, explore associations with a range of covariate factors prominent in adolescence, and establish whether subscribing to different health behaviour patterns contributes to variance in prospective mental wellbeing one year later. A three-class solution provided an excellent fit to the data, discriminating between the *Wellness Weary* (a relatively unhealthy class), the *Balanced Bunch* (a moderately healthy class), and the *Green and Dream Team* (a relatively healthy class), in alignment with H1. A large number of covariate factors were significantly associated with latent class membership. Most notably, ethnic minorities and those subject to higher levels of socio-economic disadvantage were *less* likely, and cisgender heterosexual girls were *more* likely, to be members of healthier classes. There were no observed effects of bullying victimisation on health behaviour class membership meaning findings offer partial support for H2.

In the unadjusted model, comparisons of the T2 outcomes of the three latent health behaviour classes were directly in line with our predictions, with healthier classes reporting significantly better mental wellbeing. Differences were attenuated in the fully adjusted model, with those between the *Wellness Weary* and the *Balanced Bunch* (*d* = -.019), and the *Wellness Weary* and the *Green and Dream Team* (*d* = .032) becoming non-significant. Indeed, the only effect remaining was for the *Green and Dream Team*, who had marginally better mental wellbeing than the *Balanced Bunch* (*d* = .050). Accordingly, we are only able to offer partial support for H3.

Previous studies exploring patterns of health behaviour such as physical activity or eating habits have typically identified three to seven clusters depicting healthy, unhealthy and mixed patterns [59]. Adolescent *risk*-behaviours have also been found to cluster in a similar way [60]. In one such study, three distinct patterns of risk-behaviours (binge drinking, low fruit and vegetable intake, physical inactivity, insufficient sleep, and smoking) were uncovered, and high-risk classes had significantly poorer mental health [60]. Findings from the present study concur, showing that adolescents vary substantially in the extent to which they engage in health behaviours, and that the collective effect of health behaviour patterns have potential to enhance or diminish mental wellbeing.

The emergence of distinct classes of health behaviour can be explained through the lens of Health Lifestyle Theory [61]. This theory proposes that healthy lifestyles are not the product of uncoordinated behaviours of disconnected individuals, but instead are a consequence of the complex interplay between societal structures, individual agency, group-based identities, and cultural norms [61, 62]. Whilst a young person’s free will to make healthy choices undoubtably contributes to variance in health behaviour endorsement, social and demographic factors can greatly limit or enhance the actual and perceived choices available to a specific class or subset of the population [61, 62]. For this reason, groups of individuals with similar social and demographic characteristics may encounter similar barriers or drivers that lead to the emergence of common health behavioural patterns.

### Socio-economic disadvantage

Of all covariates measured, socioeconomic disadvantage was the most strongly associated with membership of both the *Wellness Weary* and *Balanced Bunch* classes. Existing evidence concerning the impact of socio-economic variables on adolescents’ physical activity paints a mixed picture. Adolescents from disadvantaged households might rely more on active transportation if they lack access to a car but could face barriers such as unaffordable membership fees for after-school sports clubs resulting in socio-economic differences in the types, domains, and volume of physical activity they engage in [13, 63, 64]. The effects of disadvantage extend to other health behaviours with links to lower fruit and vegetable consumption [17], more sugary drinks [16] and fragmented sleep [18]. In the current study, those most likely to endorse one health behaviour (e.g., physical activity) were most likely to endorse concomitant health behaviours. This consistency in the rank order of health behaviour patterns across all latent class indicators offers further support for Health Lifestyle Theory [61, 62] and infers that underlying social disparity may have ubiquitous impact on multiple health behaviours simultaneously. Alas, the health effects of socio-economic disparity persist. The need to create a more fair and equitable society must continue to be of paramount importance to public health advocates [65].

### Further demographic considerations

Reports show a widening gap in the physical activity levels of Black and Asian adolescents compared to those from other ethnic backgrounds [13]. The current study expands knowledge in this regard, establishing that not only are Black and Asian adolescents least likely to be active, but they are also least likely to get sufficient sleep or have healthy eating habits, placing them among the least healthy generally. The health behaviour patterns of these ethnic minorities conflict with reports from both this cohort and others that Black and Asian adolescents have at least equal (if not higher) levels of mental wellbeing than many other ethnic groups [66–68]. Similarly, a plethora of sources report girls have lower physical activity levels, lower mental wellbeing and greater mental health difficulties than boys [13, 66, 69] yet were substantially more likely to be *Green and Dream Team* members. In essence, findings reveal a partial disconnect between health lifestyles and adolescent mental wellbeing. Whilst there is evidence for a longitudinal association between the two, the strength of this association is highly subject to demographic and socio-economic influences that should be factored into public health messaging and intervention/prevention strategies going forward. Moreover, disparity on the grounds of ethnicity, socio-economic position, gender, and sexual orientation is a concern regardless of any links with mental wellbeing. Evidence that over one in ten young people (see Fig. 3 for proportional distribution) are associated with a *Wellness Weary* (less physical activity, insufficient sleep, and infrequent fruit and vegetable intake) pattern of behaviour is worrying in and of itself.

### Social media use

Young people are extensive social media users, yet there is a lack of research on the effect of social media on health behaviour [70]. The current study contributes to knowledge by establishing that lower daily usage increases the probability of having more favourable health behaviour patterns. This builds on evidence linking social media use to difficulties with sleep such as delayed sleep onset and increased night-time awakenings [19]. Excessive use of social media to share or promote physical activities can contribute to exercise compulsion and pressure to conform to certain body-image standards which in turn can have negative consequences for mental wellbeing [20]. Efforts to improve health behaviours among young people might usefully focus on finding the balance (i.e., between time spent on social media and time spent engaging in health behaviours.

Positive associations have previously been observed between social media and confectionary consumption, partly attributed to marketing campaigns involving celebrities designed to target impressionable teens [71, 72]. Whilst we did not directly test whether social media use predicted confectionary consumption directly (rather, usage was related to endorsement of health behaviour patterns as a whole), there was little variation in the level of confectionary consumption across classes inferring the impact of social media did not lead to the creation of classes with meaningfully different levels of consumption.

### Health behaviours and later mental wellbeing

Before adjusting for covariates, the magnitude of effect sizes and contrasts between classes were in line with expectations (i.e., *Green and Dream Team* > *Balanced Bunch; Green and Dream Team* > *Wellness Weary*; *Balanced Bunch* > *Wellness Weary*). This pattern of findings coincides with prior research that utilised a person-centred approach to understand links between health behaviours and mental wellbeing in adolescence [35] and suggests physical activity, sleep quality and a healthy diet can support mental wellbeing, even when the resultant lifestyle remains suboptimal. The partially adjusted analysis highlights that baseline mental wellbeing accounts for a substantial proportion of the variance between all classes (Table 5), but the combined effect of all covariates (including baseline mental wellbeing) does not explain all of the variance at T2 between the *Green and Dream Team* and the *Balanced Bunch.* The persistence of a small but significant difference between these groups in the fully adjusted analysis suggests that even when additional sociodemographic factors are accounted for, health behaviours still confer slight improvements in mental wellbeing, for the most healthy over moderately healthy adolescents.

Importantly, differences in mental wellbeing between moderately healthy adolescents and those exhibiting the least healthy behaviours were not significant. It is possible this unexpected finding is related to the sociodemographic makeup of each of the classes and how differences in young peoples’ subjective perceptions of their living conditions may have an enduring effect on mental wellbeing even after accounting for the health behaviours they endorse. Relative Deprivation Theory [73] posits that when a young person perceives their material indicators of wealth (e.g., housing size, owning a car) to be equal to other families in their neighbourhood, socioeconomic deprivation may be less detrimental to mental wellbeing than if upon said comparison they were to find themselves disenfranchised or lacking. Aligning with this theory, it is possible for young people from disadvantaged or ‘left behind’ neighbourhoods [74] to have a more positive view of their circumstances and rate their mental wellbeing as favourably as those from more affluent neighbourhoods who perceive their resources to be insufficient compared to others in the surrounding area [75, 76]. Future research should seek to enhance understanding of neighbourhood effects on adolescent wellbeing to provide greater clarity in this regard. This departure of our findings from associations identified in prior studies may also reflect methodological differences. For example, our analysis was longitudinal as opposed to cross-sectional [35, 77, 78]; our focus was on mental wellbeing as opposed to mental illness [77, 79]; and our adjusted models accounted for a wide range of sociodemographic covariates, helping to remove certain biases and confounding effects compared to other studies making fewer adjustments [35, 77, 79].

### Strengths and limitations

The current study benefited from a very large sample, longitudinal dataset, and use of robust, person-centred statistical techniques. With the exception of free school meal eligibility, the demographic characteristics of the analytical sample are also largely representative of the area it was taken from (see Supplementary Material). However, there are a number of limitations that should be borne in mind. First, although the previously coined, ‘Big Three’ (physical activity, sleep, diet) were utilised, these are by no means the only health behaviours prevalent in adolescence. Notably, our dataset did not contain information about substance use (e.g., alcohol, cannabis), which becomes increasingly prevalent over the course of adolescence [80]. Such data are now being collected as part of the recent extension of the #BeeWell study in its second location [81] and so a future analysis can incorporate these health behaviours to address this limitation.

Importantly, whilst the majority of the sample were part of the *Green and Dream Team* (i.e., the healthiest class) caution is urged when describing this group as ‘healthy’. The *Green and Dream Team’s* likelihood of endorsing healthy behaviours is relative to the rest of the sample so members of this class and indeed all other classes, may still be insufficiently healthy according to national and/or international public health recommendations [82, 83]. Some measures used also endorsed an element of subjectivity (e.g., the single-item used to quantify sleep) and do not readily facilitate comparison against national averages or age-matched cohorts, making quantification of overall health by way of health behaviour endorsement difficult. However, it is the stance of #BeeWell that young peoples’ voices should be central to research and measures were chosen in consultation with a Youth Steering Group. It is therefore maintained that although the current study may be limited in its ability to classify participants as *healthy* or *unhealthy* according to external metrics, the data analysed, and findings presented herein, provide a useful insight into the lives of young people from Greater Manchester.

Finally, the first wave of data was collected in 2021, roughly one and a half years after the initial outbreak of COVID-19. Although we did not collect measures of mental wellbeing prior to this time and findings may not be directly comparable, through a collaboration with the Life Readiness Survey in Greater Manchester [84] #BeeWell has evidence that during the time of data collection for the current study, young peoples’ feelings of hope and optimism for the future were yet to return to pre-pandemic levels. The sharp decline and gradual recovery in hope and optimism in response to the pandemic is likely mirrored in their mental wellbeing across this period meaning today, young people may report minor improvements in mental wellbeing than levels reported in 2021/22. We will continue to *listen* to the voices of young people as time goes on to monitor whether this is the case, to *act* on new information as it comes offering support where needed, and work with local authorities and professional services to *celebrate* their mental wellbeing.

## Conclusions

This study identified three distinct patterns of health behaviour among adolescents, broadly categorised as more, moderate, and less healthy. The observed consistency in engaging in multiple health behaviours corroborates the tenets of Health Lifestyle Theory. Notably, disparities rooted in ethnicity and socio-economic status were evident, with minority and socio-economically disadvantaged adolescents more frequently adopting less healthy behavioural patterns. Public health initiatives must continue to focus on reducing these disparities. Additionally, curbing social media use could present a less impactful, yet more timely strategy for encouraging adherence to healthier behaviour patterns. The healthiest class of adolescents demonstrated significantly greater mental wellbeing than their moderately healthy peers, yet a considerable segment of the cohort fell into the least healthy category with whom no difference was observed. Findings collectively underscore the imperative to enhance health behaviour and combat social disparity during adolescence to foster better health outcomes.

## Supplementary Information

Supplementary Material 1: Table S1. Demographic characteristics of the analytical sample, as well as Greater Manchester and national statistics pertaining to adolescents. Table S2. Model fit statistics for latent classes of health behaviour (split halves analysis: half one). Figure S1. Elbow plot illustrating model fit (split halves analysis: half one). Figure S2. Three latent classes of adolescent health behaviour (split halves analysis: half one). Table S3. Model fit statistics for latent classes of health behaviour (split halves analysis: half two). Figure S3. Elbow plot illustrating model fit (split halves analysis: half two). Figure S4. Three latent classes of adolescent health behaviour (split halves analysis: half two). Table S4. Item response probabilities and model estimated mean scores for class indicators (split halves analysis). Table S5. Associations between latent classes of health behaviour and covariates (complete case sensitivity analysis).

## Data Availability

An anonymised version of the #BeeWell survey responses will be made publicly available in 2026. Due to ethical governance constraints this cannot be brought forward since participants have been given the right to withdraw their data until this point, necessitating the need to maintain a securely stored pseudonymised version until this point. In addition, the linked administrative data (e.g., sex, free school meal eligibility) will never be shared publicly due to the prohibition of onward sharing in the data sharing agreement in place with the Local Authorities who provided it. To request access to the #BeeWell data, please contact Neil Humphrey at neil.humphrey@manchester.ac.uk. Anonymous Mplus syntax used to analyse the data will be made publicly available via the Open Science Framework upon acceptance of this manuscript for publication.

## Abbreviations

AIC: Akaike Information Criteria
BIC: Bayesian Information Criteria
CMO: Chief Medical Officers
FIML: Full Information Maximum Likelihood
H1: Hypothesis one
H2: Hypothesis two
H3: Hypothesis three
LGBTQ+: Lesbian Gay Bisexual Transgender Queer
LL: Loglikelihood
LMRa: Lo-Mendell-Rubin Adjusted Likelihood Ratio Test
ssaBIC: Sample Size Adjusted Bayesian Information Criteria
T1: Timepoint one
T2: Timepoint two
