# Supplementary material for: Latent classes of adolescent health behaviour, social covariates and mental wellbeing: a longitudinal birth cohort study
Source: BMC Public Health. 2024 Sep 18;24:2538. doi: 10.1186/s12889-024-20004-y (PMC11409474; doi:10.1186/s12889-024-20004-y)
Supplement: Supplementary file 1 — Supplementary Material 1: Table S1. Demographic characteristics of the analytical sample, as well as Greater Manchester and national statistics pertaining to adolescents. Table S2. Model fit statistics for latent classes of health behaviour (split halves analysis: half one). Figure S1. Elbow plot illustrating model fit (split halves analysis: half one). Figure S2. Three latent classes of adolescent health behaviour (split halves analysis: half one). Table S3. Model fit statistics for latent classes of health behaviour (split halves analysis: half two). Figure S3. Elbow plot illustrating model fit (split halves analysis: half two). Figure S4. Three latent classes of adolescent health behaviour (split halves analysis: half two). Table S4. Item response probabilities and model estimated mean scores for class indicators (split halves analysis). Table S5. Associations between latent classes of health behaviour and covariates (complete case sensitivity analysis). [file 12889_2024_20004_MOESM1_ESM.docx]

**Latent Classes of Adolescent Health Behaviour, Social Covariates and Mental Wellbeing: A Longitudinal Birth Cohort Study (Supplementary Material)**

Table S1: Demographic characteristics of the analytical sample, Greater Manchester (GM) and national population of adolescents

|  | Sex (%) | FSM (%) | EAL (%) | SEN (%) | Ethnicity (%) |
| --- | --- | --- | --- | --- | --- |
| Analytical Sample | F = 50.5  M = 49.4  NA = 0.1 | No = 72.2  Yes = 25.6  NA = 2.2 | No = 78.0  Yes = 20.7  NA = 1.3 | No = 83.7  Yes = 14.9  NA = 1.5 | AOEG = 2.2  Asian = 16.9  Black = 4.8  Chinese = 0.9  Mixed = 5.8  Unclassified = 2.0  White = 64.0  NA = 3.4 |
| GM Population of Year 8 Pupils^a^ | F = 48.7  M = 51.3 | No = 64.4  Yes = 35.6 | ^*^No = 76.9  ^*^Yes = 22.7  ^*^Unclassified = 0.4 | No = 81.1  Yes = 18.9 | ^*^AOEG = 3.0  ^*^Asian = 17.6  ^*^Black = 7.1  ^*^Chinese = 1.1  ^*^Mixed = 6.5  ^*^Unclassified = 1.7  ^*^White = 63.1 |
| National Statistics for Year 8 Pupils^a^ | F = 48.7  M = 51.3 | No = 71.0  Yes = 29.0 | ^*^No = 81.2  ^*^Yes = 18.1  ^*^Unclassified = 0.7 | No = 81.5  Yes = 18.5 | ^*^AOEG = 2.3  ^*^Asian = 12.2  ^*^Black = 6.3  ^*^Chinese = 0.7  ^*^Mixed = 6.5  ^*^Unclassified = 2.0  ^*^White = 70.0 |

EAL *English as an additional language;* FSM *Free School Meal Eligibility over the last six years;* SEN *Special Educational Needs*

^a^*National statistics data gathered in the academic year 2022/23:* [*here*](https://explore-education-statistics.service.gov.uk/find-statistics/school-pupils-and-their-characteristics)

^*^*Proportions for Year 8 were unavailable so statistics represent proportions across years in state-funded secondary schools*

Table S2: Model fit statistics for latent classes of health behaviour (half one)

| Classes | LL | AIC | BIC | ssaBIC | LMRa | Entropy | Model Estimated Class Proportions |
| --- | --- | --- | --- | --- | --- | --- | --- |
| 1 | -44973.693 | 89959.386 | 90002.173 | 89983.106 | - | - | 1 |
| 2 | -44162.679 | 88347.359 | 88425.802 | 88390.846 | .000 | .739 | .42, .58 |
| 3 | -43353.928 | 86739.855 | 86853.954 | 86803.109 | .000 | .916 | .40, .14, .46 |
| 4 | -43240.145 | 86522.290 | 86672.045 | 86605.311 | .000 | .836 | .46, .16, .27, .11 |
| 5 | -40013.428 | 80078.856 | 80264.267 | 80181.643 | .000 | .995 | .14, .16, .25, .24, .21 |

*Note: only two random starts converged for the 5-class solution so could indicate non-convergence.*

*
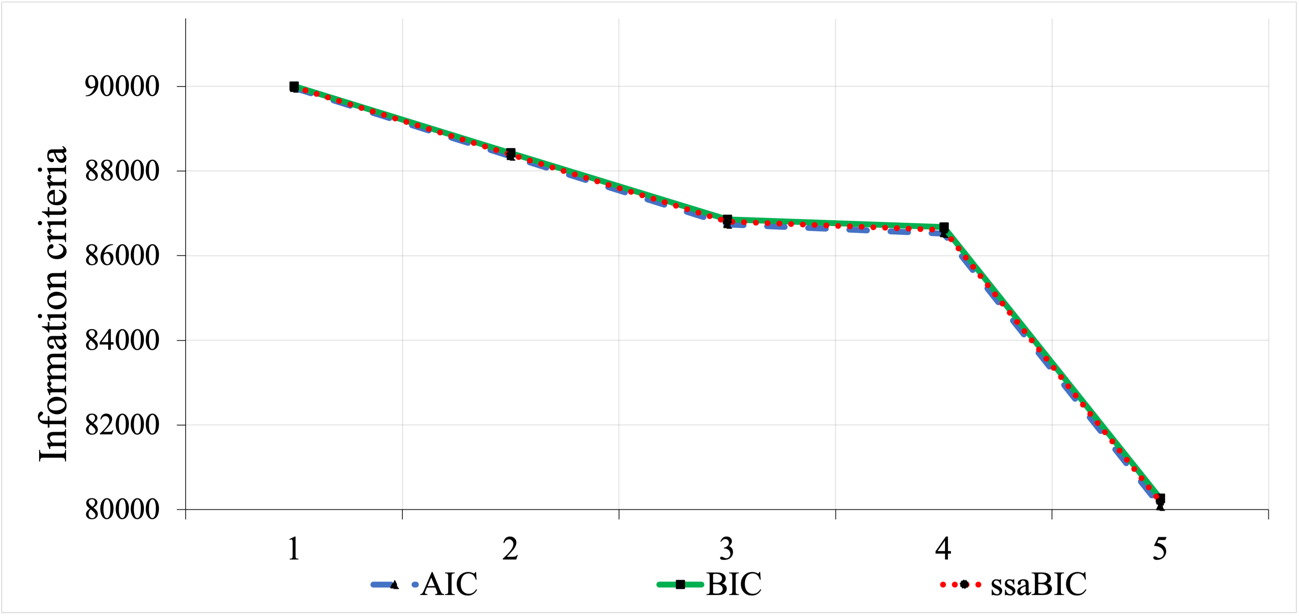
*Figure S1: Elbow plot illustrating model fit (half one)


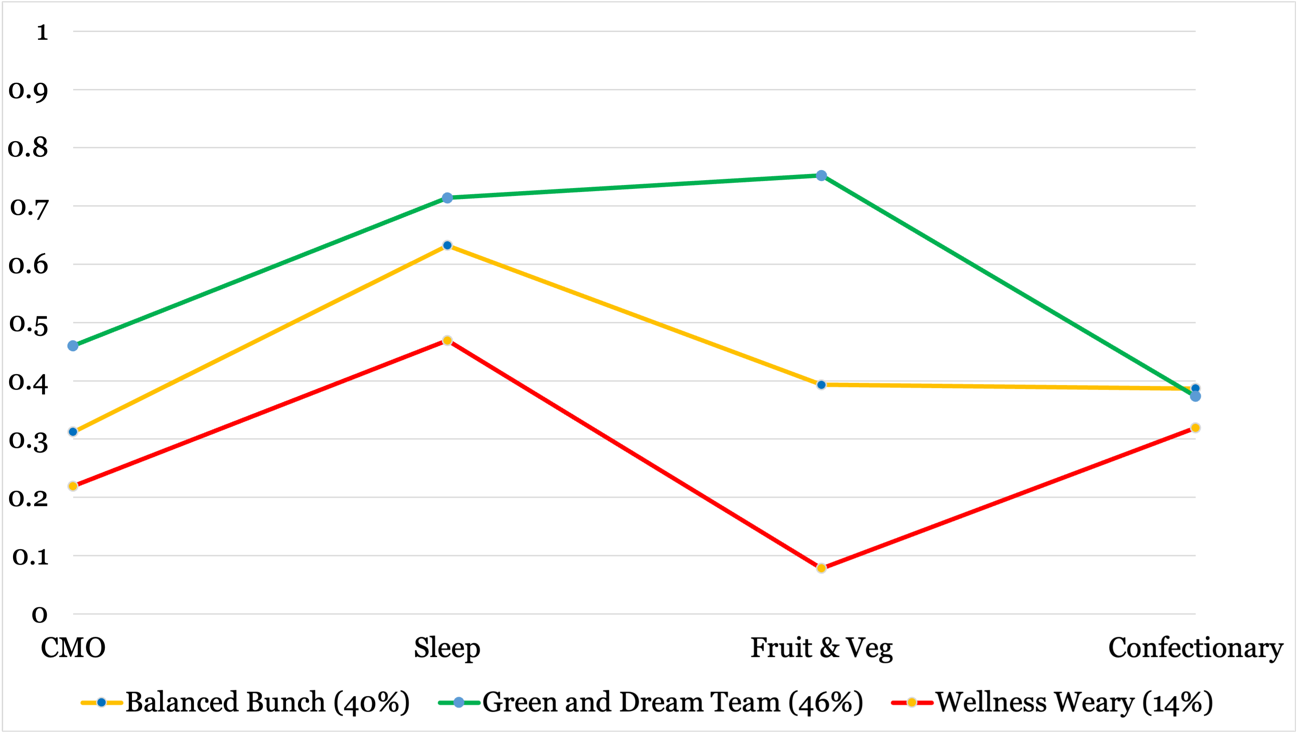
Figure S2: Three latent classes of adolescent health behaviour (half one)

Table S3: Model fit statistics for latent classes of health behaviour (half two)

| Classes | LL | AIC | BIC | ssaBIC | LMRa | Entropy | Model Estimated Class Proportions |
| --- | --- | --- | --- | --- | --- | --- | --- |
| 1 | -45231.249 | 90474.499 | 90517.286 | 90498.219 | - | - | 1 |
| 2 | -44464.455 | 88950.910 | 89029.353 | 88994.397 | .000 | .720 | .43, .57 |
| 3 | -43652.041 | 87336.083 | 87450.182 | 87399.336 | .000 | .916 | .40, .15, .45 |
| 4 | -43523.660 | 87089.320 | 87239.075 | 87172.340 | .000 | .836 | .12, .16, .45, .27 |
| 5 | -43437.672 | 86927.343 | 87112.754 | 87030.130 | .000 | .834 | .04, .16, .07, .45, .28 |


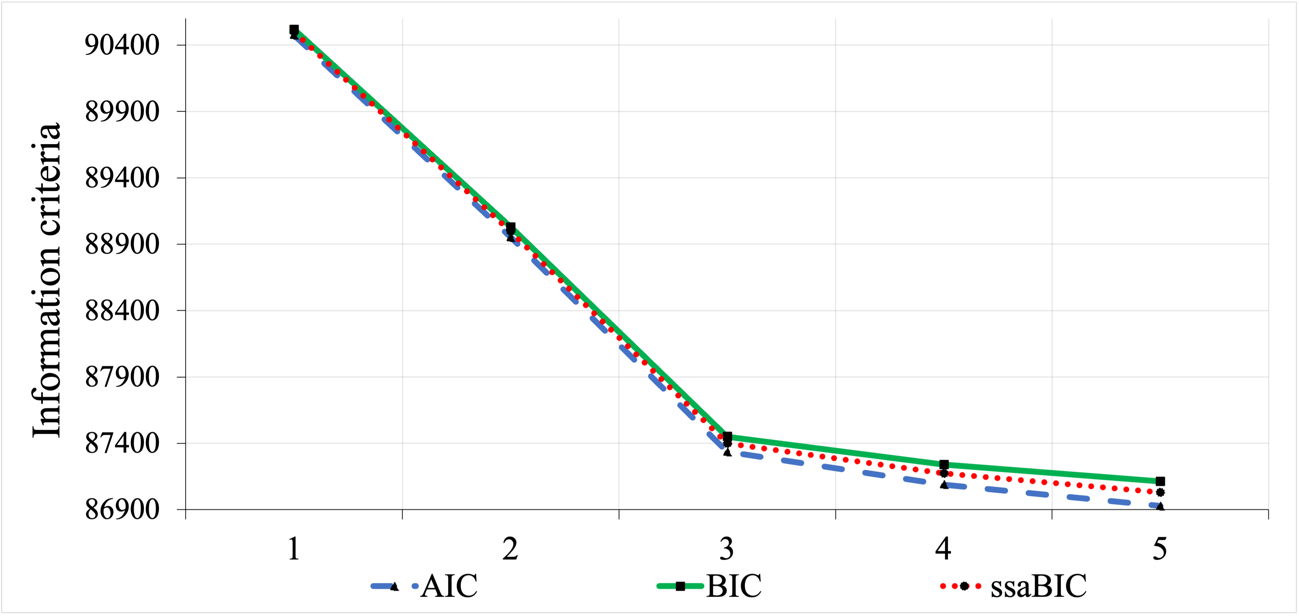
Figure S3: Elbow plot illustrating model fit (half two)


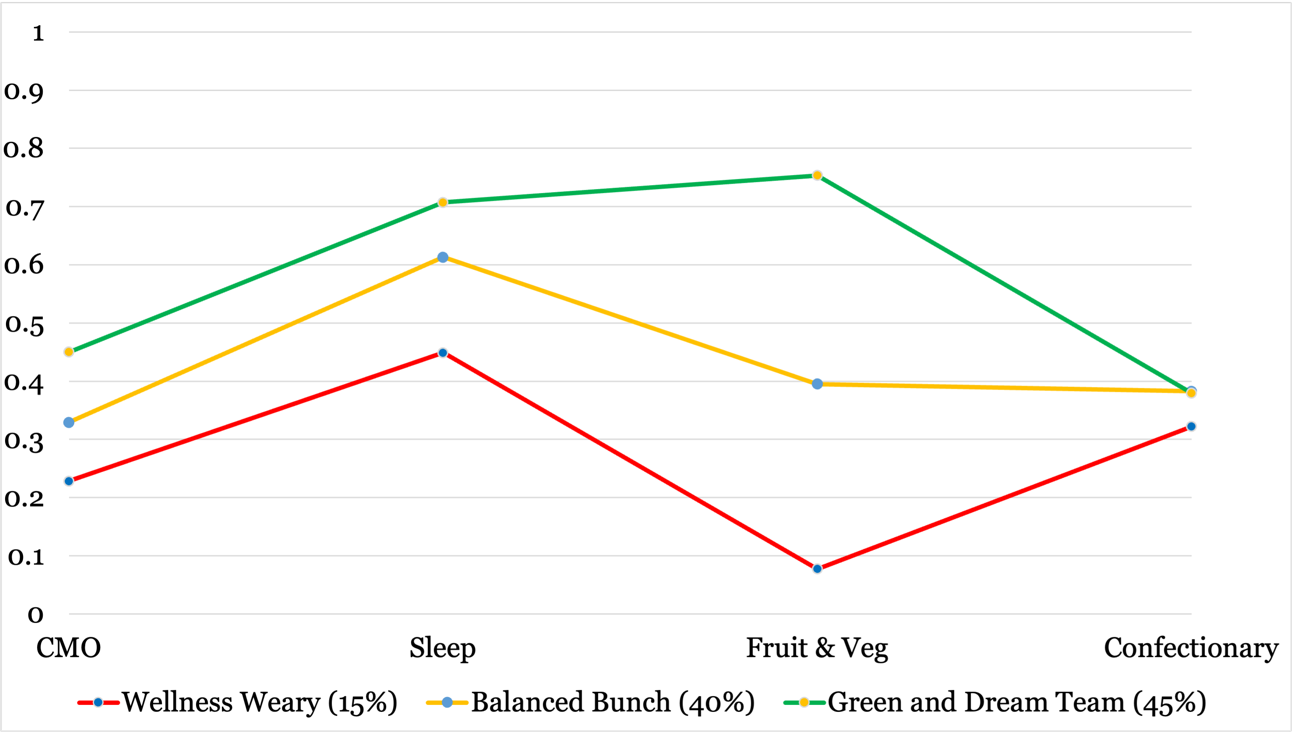
Figure S4: three latent classes of adolescent health behaviour (half two)

Table S4: Item response probabilities and model estimated mean scores for class indicators (split halves analysis)

|  | Full Sample | | | Half One | | | Half Two | | |
| --- | --- | --- | --- | --- | --- | --- | --- | --- | --- |
| Item | GDT | BB | WW | GDT | BB | WW | GDT | BB | WW |
| CMO | .455 | .320 | .224 | .460 | .312 | .219 | .450 | .329 | .228 |
| Sleep | .710 | .623 | .459 | .714 | .632 | .469 | .707 | .613 | .449 |
| Fruit and Veg | 5.517 | 3.364 | 1.466 | 5.513 | 3.358 | 1.469 | 5.520 | 3.370 | 1.464 |
| Confectionary | 3.258 | 3.308 | 2.924 | 3.238 | 3.320 | 2.916 | 3.278 | 3.295 | 2.932 |

Table S5: Associations between latent classes of health behaviour and covariates (complete case sensitivity analysis)

|  |  |  | 95% CI | |  |  |  | 95% CI | |
| --- | --- | --- | --- | --- | --- | --- | --- | --- | --- |
| Variable | Class | OR (S.E.) | Lower | Upper | Variable | Class | OR (S.E.) | Lower | Upper |
| Physical | WW | 1 | 1 | 1 | Mixed | WW | 1 | 1 | 1 |
| Health | BB | 1.232 (.045)* | 1.148 | 1.322 |  | BB | 1.240 (.150)^a^ | .978 | 1.571 |
|  | GDT | 1.590 (.058)* | 1.480 | 1.708 |  | GDT | 1.164 (.137) | .924 | 1.465 |
|  |  |  |  |  |  |  |  |  |  |
| IMD | WW | 1 | 1 | 1 | AOEG | WW | 1 | 1 | 1 |
|  | BB | .590 (.075)* | .460 | .757 |  | BB | 1.223 (.340) | .709 | 2.110 |
|  | GDT | .298 (.043)* | .225 | .395 |  | GDT | 1.945 (.582)*^a^ | 1.083 | 3.496 |
|  |  |  |  |  |  |  |  |  |  |
| Social | WW | 1 | 1 | 1 | Cisgender | WW | 1 | 1 | 1 |
| Media | BB | .931 (.012)* | .907 | .955 | Heterosexual | BB | 1.223 (.101)* | 1.041 | 1.438 |
|  | GDT | .845 (.012)* | .822 | .869 | Girl | GDT | 1.493 (.128)* | 1.262 | 1.767 |
|  |  |  |  |  |  |  |  |  |  |
| Bullied | WW | 1 | 1 | 1 | LGBTQ+ | WW | 1 | 1 | 1 |
|  | BB | .887 (.069) | .761 | 1.033 |  | BB | .827 (.065)* | .709 | .964 |
|  | GDT | .993 (.080) | .848 | 1.163 |  | GDT | .969 (.079) | .827 | 1.136 |
|  |  |  |  |  |  |  |  |  |  |
| Asian | WW | 1 | 1 | 1 | SWEMWBS | WW | 1 | 1 | 1 |
|  | BB | .830 (.071)* | .702 | .981 |  | BB | 1.043 (.008)* | 1.028 | 1.059 |
|  | GDT | .789 (.070)* | .664 | .937 |  | GDT | 1.063 (.007)* | 1.049 | 1.078 |
|  |  |  |  |  |  |  |  |  |  |
| Black | WW | 1 | 1 | 1 |  |  |  |  |  |
|  | BB | .565 (.094)* | .407 | .784 |  |  |  |  |  |
|  | GDT | .498 (.072)* | .374 | .662 |  |  |  |  |  |

* indicates significant ORs (95% CIs do not cross 1)

^a^ Cases where discrepancies in statistical significance were observed between FIML and complete case analysis
